# Correlation between intrahepatic iodine quantity after portal vein embolization and future liver remnant hypertrophy

**DOI:** 10.1007/s11604-025-01906-2

**Published:** 2025-11-10

**Authors:** Tomohiro Komada, Yuki Sato, Masaya Matsushima, Takeshi Uemura, Daiki Tamashiro, Ryota Asai, Kyoko Ito, Takashi Mizuno, Tomoki Ebata, Shinji Naganawa

**Affiliations:** 1 Department of Radiology, Nagoya University Graduate School of Medicine, 65 Tsurumai, Showa-Ku, Nagoya, Aichi 466-8550 Japan; 2 Canon Medical Systems Corporation, Otawara, Japan; 3 Division of Surgical Oncology, Department of Surgery, Nagoya University Graduate School of Medicine, Nagoya, Japan

**Keywords:** Hepatectomy, Hypertrophy, Liver, Portal vein, X-ray computed tomography

## Abstract

**Purpose:**

This study investigated the correlation between future liver remnant (FLR) hypertrophy and iodine content in the FLR following portal vein embolization, as measured by dual-energy computed tomography (DECT) with direct injection of the contrast agent into the portal vein.

**Materials and methods:**

In this prospective study conducted at a single center, 39 patients with biliary tract carcinoma underwent right portal vein embolization prior to extended hepatectomy. After portal vein embolization, DECT was performed after injecting iodinated contrast medium into the portal vein, allowing the quantification of iodine concentration and iodine content in the FLR. Liver volumes were assessed before and after portal vein embolization to calculate the degree of hypertrophy and kinetic growth rate of the FLR. Correlations between iodine content and hypertrophy indices, such as degree of hypertrophy and kinetic growth rate, were analyzed.

**Results:**

Post-portal vein embolization volumetric CT for FLR hypertrophy evaluation was performed at a median of 25 days (21–30 days). Early-phase iodine content of the left hepatic lobe on DECT was significantly correlated with both the degree of hypertrophy (DH; r = 0.380, *p* = 0.038) and the kinetic growth rate (KGR; r = 0.401, *p* = 0.028), as determined using Pearson’s correlation analysis. Likewise, late-phase iodine content of the left hepatic lobe was significantly correlated with DH (r = 0.403, *p* = 0.011) and KGR (r = 0.337, *p* = 0.036).

**Conclusions:**

Higher iodine content in the FLR was associated with greater FLR hypertrophy after portal vein embolization. However, because the correlation observed in this study was modest, the predictive ability of this parameter could not be established. Nevertheless, DECT-based iodine quantification may provide complementary information on FLR function and warrants further investigation as a potential biomarker in future studies.

## Introduction

Complete surgical resection is considered the optimal curative approach for biliary carcinoma [1–3]. However, major hepatectomies often require the removal of large portions of liver parenchyma, potentially resulting in postoperative liver failure if the future liver remnant (FLR) has insufficient volume [4, 5]. To mitigate this risk, portal vein embolization (PVE) is commonly performed before extensive liver resection, as embolizing the portal branches supplying the liver segments planned for resection directs portal blood flow towards the segments to be preserved, thereby stimulating compensatory hypertrophy of the FLR [6, 7]. Despite its widespread use, the degree of FLR hypertrophy after PVE varies considerably. Various predictors of FLR hypertrophy have been investigated, including patient demographics and the types of embolic materials used [8–11]. However, directly measuring the portal venous flow that contributes to FLR hypertrophy remains challenging, as the portal vein is located deep within the body.

Dual-energy computed tomography (DECT) allows the quantification of tissue iodine concentrations by acquiring images at different X-ray energies. In clinical practice, DECT has demonstrated greater diagnostic accuracy than conventional CT for pulmonary embolism by enhancing the visualization of iodine contrast distribution in the lung parenchyma [12]. In hepatic imaging, DECT has also been utilized to assess liver fibrosis and micro-invasion of hepatocellular carcinoma [13–15]. As the contrast agent in contrast-enhanced DECT examinations is usually injected via a peripheral vein (e.g., from the arm), it first reaches the liver through the hepatic artery. Consequently, when the contrast agent reaches the liver parenchyma via the portal vein from the intestines, hepatic arterial enhancement is already present, making it impossible to evaluate the isolated contribution of portal venous flow. An imaging method utilizing only portal vein blood flow is CT during arterial portography. This technique is also useful for detecting liver tumors, but it requires arterial puncture, making it an invasive examination [16]. If iodinated contrast media are directly injected into the portal vein during PVE, DECT can objectively and quantitatively evaluate the distribution of portal vein flow within the liver by accurately quantifying the distribution of iodinated contrast media in the liver parenchyma without adding invasiveness.

We hypothesized that by injecting an iodine contrast agent through a catheter placed in the portal vein immediately after PVE, acquiring DECT images, generating an iodine map, and measuring the iodine content of the FLR, we could evaluate the degree of portal perfusion in the FLR. Since portal perfusion is a key factor influencing subsequent FLR hypertrophy, DECT-based iodine quantification may serve as an objective imaging parameter reflecting this physiological process. Exploring this relationship could provide valuable insights and contribute to the development of novel imaging biomarkers in future studies.

This prospective study investigated the correlation between FLR hypertrophy and iodine content in the FLR after PVE, as measured by DECT with contrast medium injected directly into the portal vein.

## Materials and methods

The Ethics Review Committee of Nagoya University Graduate School of Medicine (Nagoya, Japan) approved this prospective single-center study (approval no. 2021–0299). All participants provided written informed consent for both research and intervention. This study was reported in line with the Strengthening the Reporting of Observational Studies in Epidemiology (STROBE) criteria.

### Participants

Candidates were selected among patients scheduled for PVE prior to extended hepatectomy at our hospital, based on the following criteria: (1) having biliary tract carcinoma, (2) being scheduled for embolization of the right portal vein branch prior to right caudate liver lobectomy, and (3) having provided prior written consent to participate in the study. Patients who experienced any of the following situations were excluded: (1) technical issues during CT imaging that delayed the timing of the contrast agent, (2) preferential injection of a high concentration of contrast agent into the vessels of the caudate lobe from the catheter side hole that resulted in reduced enhancement of the left hepatic lobe, (3) mechanical issues preventing dual-energy imaging, (4) discontinuation of the PVE procedure, and (5) change in the PVE site.

Using G*Power 3.1.9.7 (https://www.psychologie.hhu.de/arbeitsgruppen/allgemeine-psychologie-und-arbeitspsychologie/gpower), we calculated that a minimum sample size of 26 participants was required based on an effect size of 0.5, an α level of 0.05, and a power (1-β) of 0.8.

### PVE technique

PVE was performed percutaneously and transhepatically via the ipsilateral approach. The embolic materials included gelatin sponge particles (Serescue; Astellas, Tokyo), followed by metallic coils (MReye Embolisation Coil; Cook Inc., Bloomington, IN) placed proximally to achieve complete embolization of the right portal vein branch. This embolization technique has been employed at our institution for over 10 years, with confirmed safety and efficacy for inducing FLR hypertrophy [17]. In some cases, small proximal branches remained patent at the operator’s discretion. Portal vein branches supplying the caudate lobe were not embolized.

### DECT protocol for portal iodine measurement

**Fig. 1:**
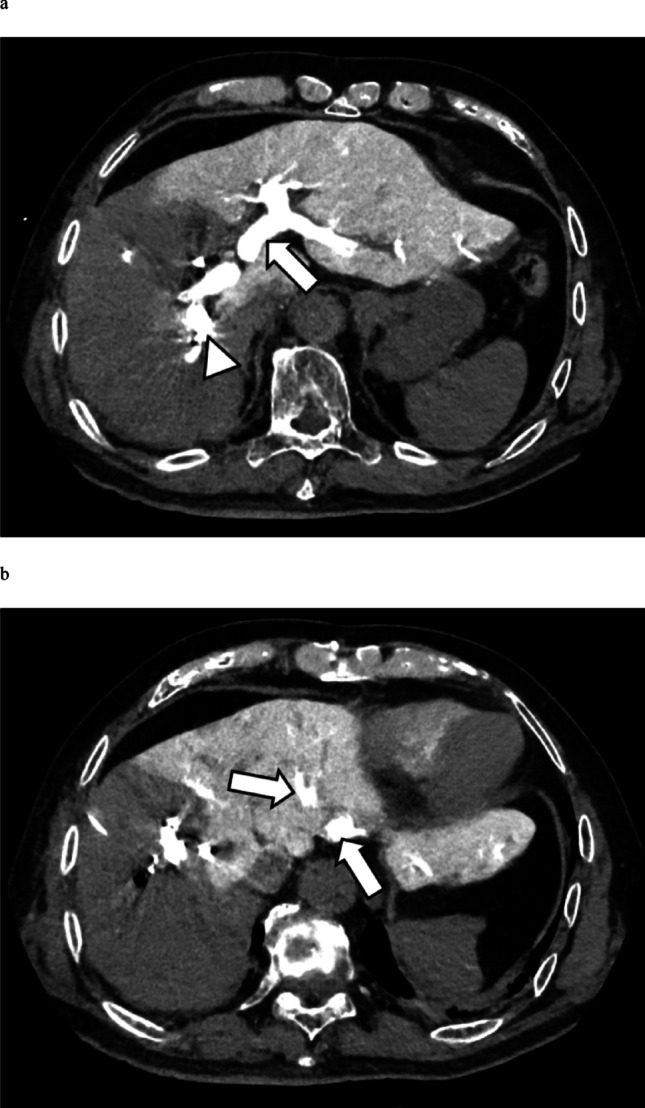
Immediately following PVE, contrast medium was injected via a catheter placed in the portal vein, and DECT imaging of the liver was performed. **a** Image acquired 6 s after contrast injection. Following embolization of the right portal vein branch, enhancement is confined to the left hepatic lobe and caudate lobe. The portal vein containing the injected contrast medium is clearly visualized (arrow). The arrowhead indicates the metallic coils. **b** Image acquired 15 s after contrast injection. While the contrast medium within the portal vein demonstrates washout, the hepatic veins (arrow) are distinctly visualized. DECT, dual-energy computed tomography; PVE, portal vein embolization

PVE and DECT imaging were performed sequentially in the same session. After PVE, while leaving the percutaneously and transhepatically inserted sheath in place, a 4-F pigtail catheter (Performa; Merit Medical Systems Inc., Salt Lake City, UT) was positioned in the main portal vein. Iodinated contrast medium was then injected through this catheter, and the liver was imaged using a hybrid interventional radiology/CT system equipped with DECT based on a 320-detector row scanner (Aquilion ONE/Nature Edition; Canon Medical Systems, Tochigi). In total, 60 mL of a 1:5-diluted iodine contrast medium (iopamidol 370 mgI/mL, Iopamiron injection 370; Bayer Yakuhin, Ltd, Osaka) was injected at a rate of 5 mL/s, and DECT was performed. The parameters for interventional radiology/CT were as follows: tube voltage, rapid kV-switching (135 and 80 kV); tube current, CT-automatic exposure control (standard deviation, 3.5; volume CT dose index, 8.6 ± 1.2 mGy); collimation, 320 × 0.5 mm; rotation time, 0.5 s/rotation; and reconstruction, deep learning-based spectral reconstruction (Spectral Body Standard). Spectral Body, an image reconstruction processing technology developed via deep learning, had no self-learning function. Two scans were initially performed: an early phase (6 s after injection) and a late phase (15 s after injection). The early phase was timed to capture the contrast medium while it remained in the portal vein, and the late phase was obtained after the contrast had cleared from the portal vein and reached the hepatic veins (Fig. 1).

### Iodine concentration measurement

**Fig. 2:**
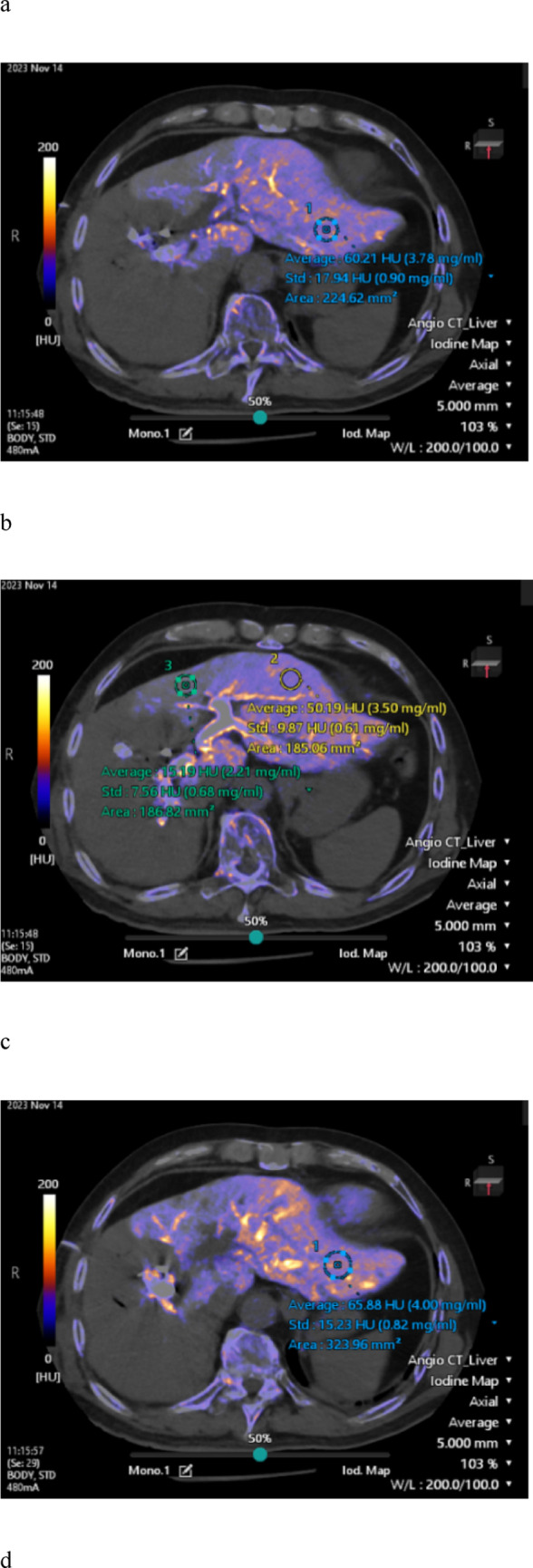
A 72-year-old male patient with perihilar cholangiocarcinoma underwent DECT, from which iodine maps were generated. Iodine concentrations were measured in both the early and late phases using ROIs larger than 1 cm^2^, placed in each segment of the left hepatic lobe (S2, S3, and S4). In the iodine map images, **a** shows a representative ROI placed in segment S2 during the early phase, whereas **b** shows representative ROIs placed in segments S3 and S4 during the early phase. Similarly, **c** shows a representative ROI in segment S2 during the late phase, and **d** shows representative ROIs in segments S3 and S4 during the late phase. DECT, dual-energy computed tomography; ROI, region of interest

Iodine maps were generated from the DECT data using workstation A (Vitrea; Canon Medical Systems), which is the standard application provided with the DECT system. To evaluate the iodine concentration in the left hepatic lobe, two radiologists (M.M. and R.A., with 20 and 2 years of experience in liver imaging, respectively) placed regions of interest (ROIs) of at least 1 cm^2^ in each hepatic segment—S2, S3, and S4—at locations considered representative of the iodine concentration in that specific segment, carefully avoiding vascular structures and ensuring the inclusion of hepatic parenchyma only (Fig. 2). Both readers measured the iodine concentration separately in each segment, and the average of their measurements was used. The iodine concentration of the left hepatic lobe was defined as the mean of the three segmental values.

**Fig. 3:**
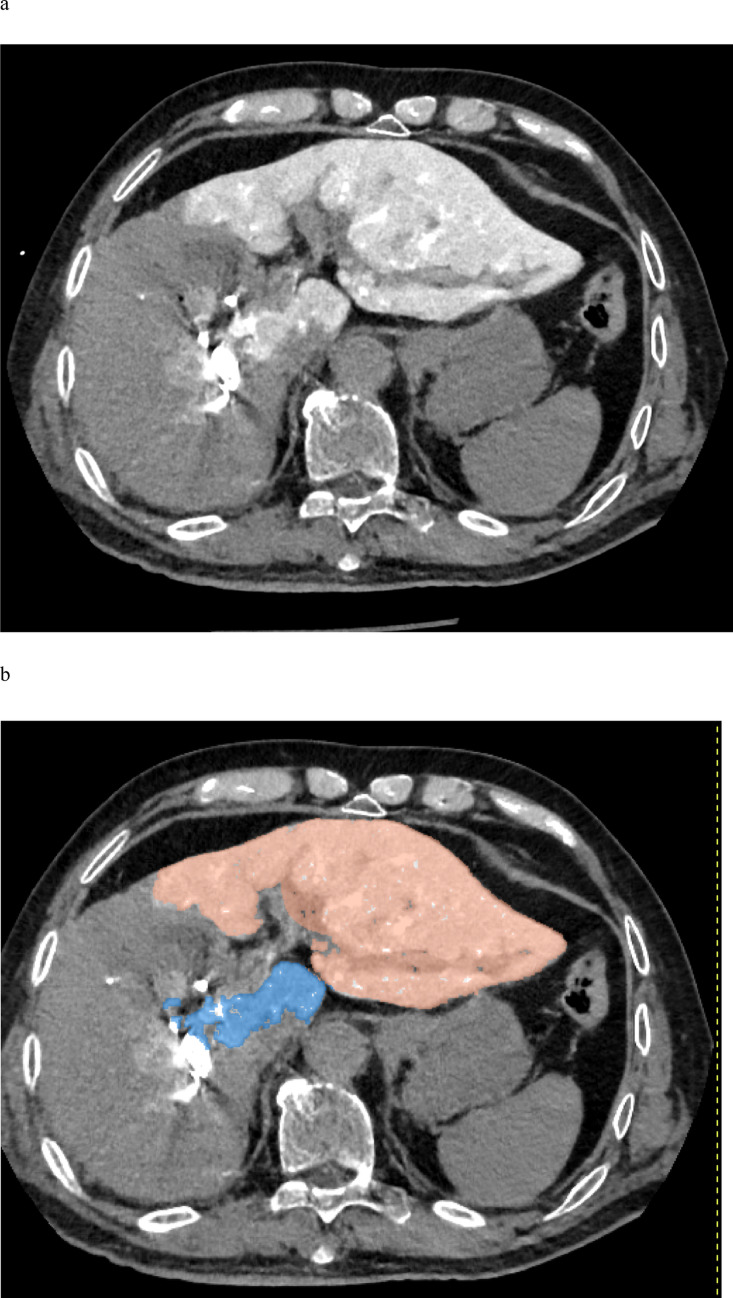
After PVE, iodine contrast medium was injected through a catheter placed in the portal vein, and the liver was imaged using a hybrid interventional radiology/computed tomography system equipped with DECT. The acquired DECT image of the liver during portal venography is shown in (**a**). Using workstation B, the total volume of the contrast-enhanced liver parenchyma was first extracted. Then, non-FLR regions—such as the caudate lobe—were excluded to isolate and measure the contrast-enhanced volume of the left hepatic lobe, corresponding to the FLR. The extracted FLR volume is illustrated in (**b**). DECT, dual-energy computed tomography; FLR, future liver remnant; PVE, portal vein embolization

To account for the intrahepatic distribution of iodine, another radiologist (T.K.; 20 years of experience in liver imaging) used a dedicated three-dimensional workstation B (Synapse Vincent; Fujifilm Medical Co., Tokyo) to extract the contrast-enhanced liver volume from late-phase DECT images obtained during portal venography. Only the volume of the FLR, corresponding to the left hepatic lobe, was extracted, whereas regions outside the left lobe (e.g., the caudate lobe) were excluded (Fig. 3). The extracted volume data were subsequently reviewed by the radiologist (M.M.).

Finally, the total iodine content in the left hepatic lobe was calculated as the product of the mean iodine concentration and the contrast-enhanced volume of the left hepatic lobe, using the following formula:

$$\begin{aligned} {\mathrm{Iodine}} {\text{content in}} {\mathrm{the}} {\text{left lobe}} \left( {mg} \right) = & {\mathrm{Mean}} {\mathrm{iodine}} {\mathrm{concentration}} {\mathrm{of}} \\ & \quad {\text{segments }}S2, S3, and S4 \left( {\frac{{{\mathrm{mg}}}}{{{\mathrm{mL}}}}} \right) \\ & \quad \times {\mathrm{Contrast}} - {\text{enhanced volume}} \\ & \quad {\mathrm{of}} {\mathrm{the}} {\mathrm{lef}}t {\mathrm{hepatic}} {\text{lobe }}\left( {{\mathrm{cm}}^{3} } \right) \\ \end{aligned}$$

### Liver volumetry before and after PVE

**Fig. 4:**
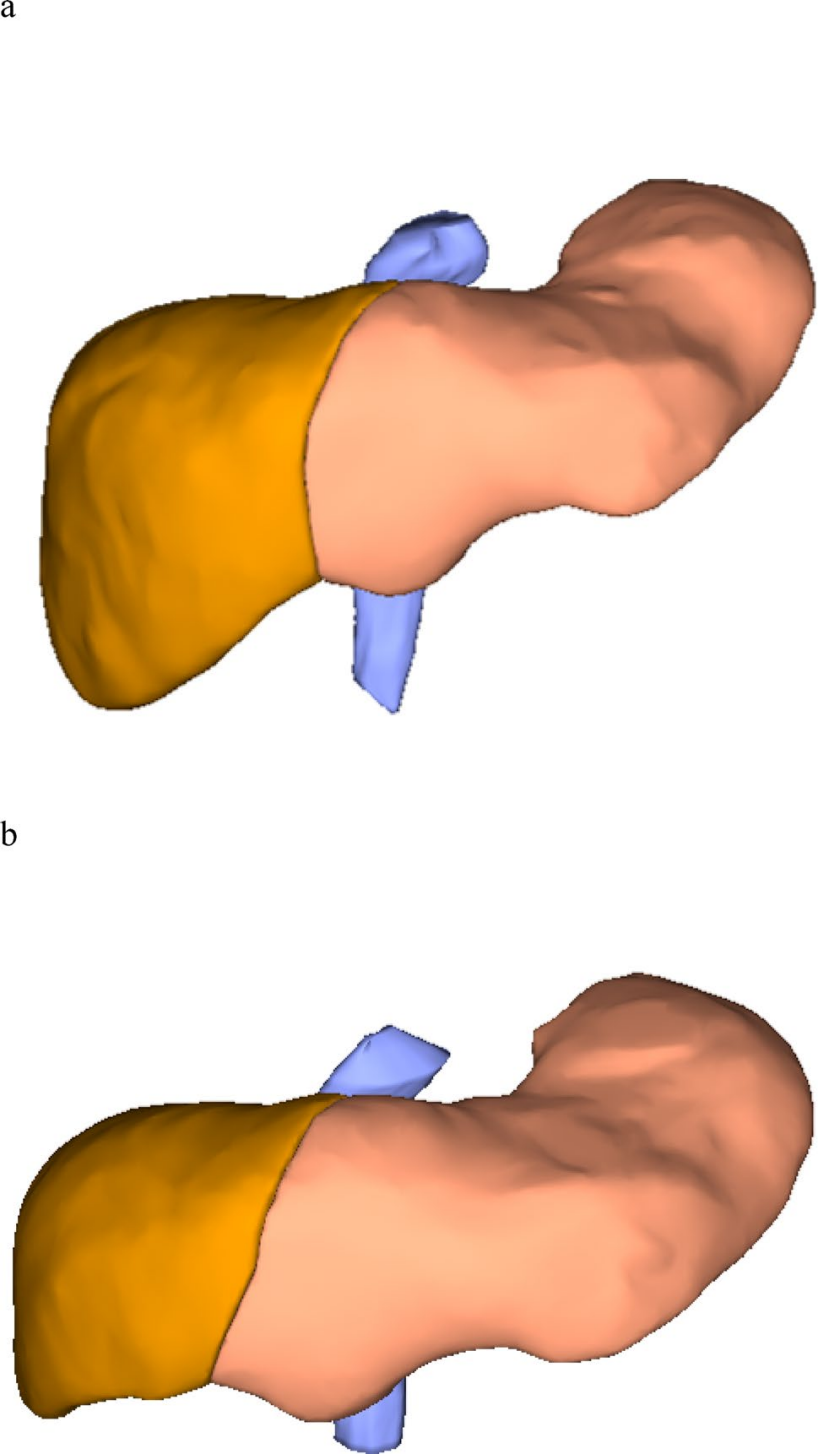
A 72-year-old male patient with perihilar cholangiocarcinoma. Liver volumes were measured using CT and a workstation before and after PVE. **a** Before PVE, the volume of the left liver lobe is 601 cm^3^. **b** After PVE, the volume of the left liver lobe has increased to 764 cm^3^. CT, computed tomography; PVE, portal vein embolization

Liver volumes were measured semi-automatically by a single radiologist (T.K.) using contrast-enhanced CT datasets acquired with ≥ 64-detector row scanners before and after PVE. Thin-slice images (≤ 2 mm) were processed on workstation B using specialized liver volumetry software. The measured volumes included both the total liver volume and the volume of the left hepatic lobe, defined as the FLR (Fig. 4). Additionally, the body surface area (BSA) was calculated from the patient’s height and weight using DuBois’ formula, and the standardized total liver volume (sTLV) was then calculated from the patient’s BSA as follows [18]:

sTLV =  − 794.41 + 1267.28 × BSA (m^2^).

Using these values, we calculated the degree of hypertrophy (DH) and the kinetic growth rate (KGR), as described in previous studies [19, 20]. Because the interval between PVE and post-PVE CT examination varied among patients, we calculated the KGR to normalize the volumetric increase per week, thereby allowing a standardized comparison of liver hypertrophy.

These indices were defined as:

$${\text{DH }}(\% ) = \frac{\begin{gathered} {\text{Left lobe volume after PVE }} - \hfill \\ \;{\text{Left lobe volume before PVE}} \hfill \\ \end{gathered} }{{{\text{ sTLV}}}}\; \times \;100$$

$${\text{KGR }}(\% {\mathrm{/week}}) = \frac{{{\mathrm{DH}}}}{\begin{gathered} {\text{Interval }}[{\mathrm{weeks}}]{\text{ between PVE and post}} \hfill \\ \; - {\text{PVE CT volume assessment}} \hfill \\ \end{gathered} }$$

### Statistical analyses

Statistical analyses were performed using SPSS Statistics for Windows (version 29; IBM Corp., Armonk, NY, USA). Categorical variables are presented as frequencies and percentages. Continuous variables are expressed as mean ± standard deviation, if normally distributed, and as median with interquartile range, if non-normally distributed. The Shapiro–Wilk test was used to assess the normality of continuous variables. As the normality was not rejected, Pearson’s correlation coefficient was employed to evaluate the associations of iodine content in the left hepatic lobe with DH and KGR. Interobserver agreement for measurement of iodine concentration in the left hepatic lobe was assessed using Spearman’s rank correlation coefficient.

To identify factors associated with left lobe hypertrophy, patients were dichotomized according to the median percentage increase in the left lobe volume (27.9%). Continuous variables were compared using the Mann–Whitney U test, and categorical variables were analyzed using Fisher’s exact test.

Postoperative liver failure was assessed according to the International Study Group of Liver Surgery (ISGLS) criteria. Patients were stratified into two groups based on the presence or absence of liver failure, and iodine content of the left lobe was compared between the groups using Student’s *t*-test.

A *p*-value < 0.05 was considered statistically significant.

## Results

### Participant characteristics

**Fig. 5:**
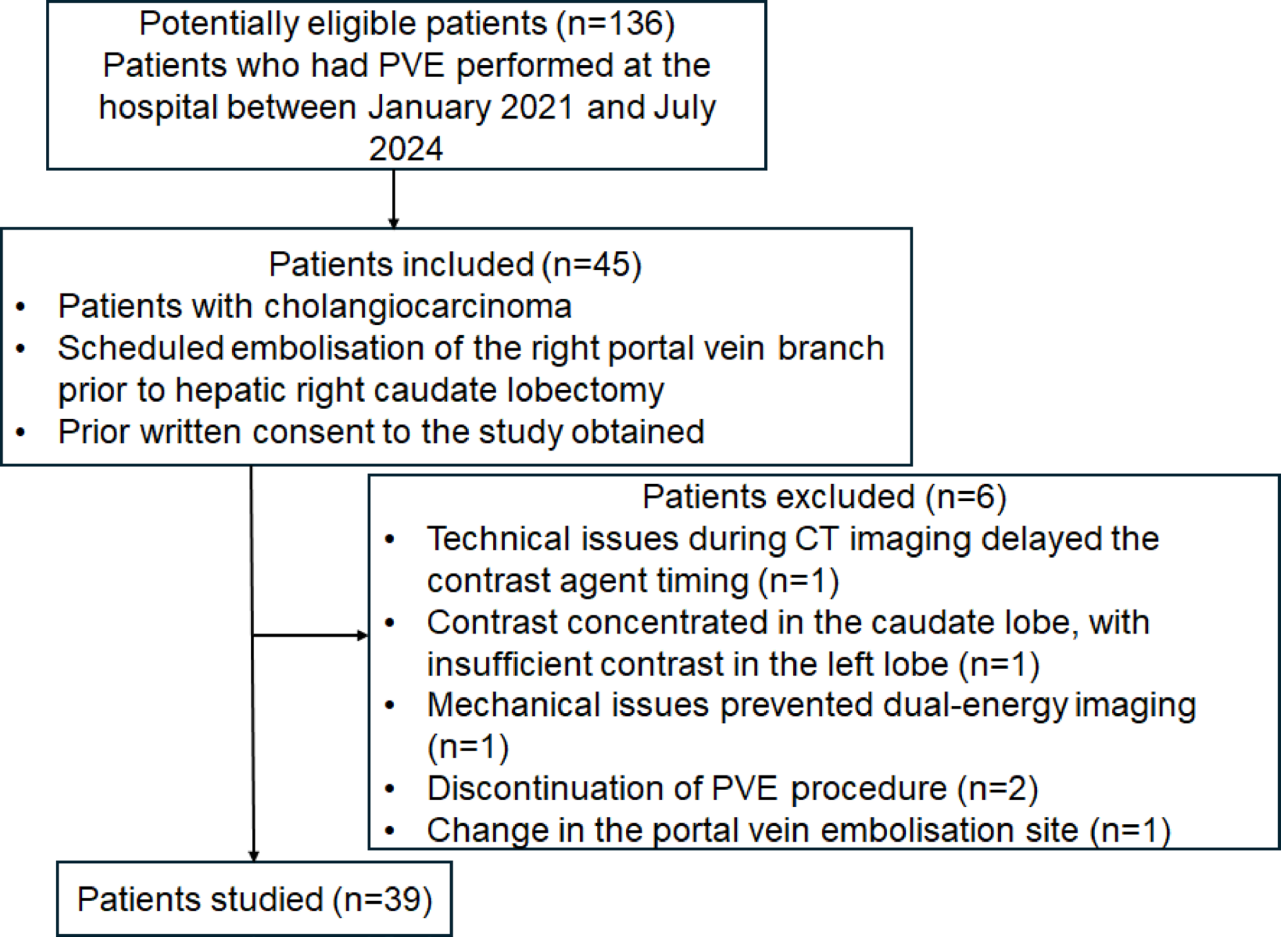
Flowchart presenting the study inclusion and exclusion criteria. CT, computed tomography; PVE, portal vein embolization

Between January 2021 and July 2024, 136 patients underwent PVE at our institution, of whom 45 patients met the inclusion criteria for this study. Seven patients were subsequently excluded based on the predefined exclusion criteria, leaving 39 participants in the final analysis. Figure 5 summarizes the patient selection process.

Table 1 presents the clinical characteristics of the included patients. Post-PVE volumetric CT for the evaluation of FLR hypertrophy was performed at a median of 25 days (21–30 days). During the study period, the DECT protocol was modified; hence, nine patients underwent only late-phase imaging, whereas the remaining 30 patients underwent both early- and late-phase imaging.

**Table 1:** Characteristics of the study participants

Parameter (n = 39)	Value
Age (y)	72 ± 8
Male, female	30 (77), 9 (23)
Body mass index (kg/m^2^)	21.9 ± 3.6
Body surface area (m^2^)	1.60 ± 0.18
Underlying liver disease	
Fatty liver	1 (3)
Preoperative chemotherapy	6 (15)
Gemcitabine + Cisplatin + Durvalumab	3 (8)
Gemcitabine + Cisplatin + S-1	2 (5)
Gemcitabine + Cisplatin	1 (3)
Laboratory data	
Total bilirubin level (mg/dL)	0.8 (0.7–1.3)
Albumin level (g/dL)	3.6 (3.3–3.9)
Platelet count (× 10^3^/μL)	217 (173–285)
Prothrombin time (%)	99.7 (93.9–104.7)
Interval between PVE and volumetric CT (d)	25 (21–30)
Final diagnosis	
Perihilar cholangiocarcinoma	34 (87)
Gallbladder carcinoma	4 (10)
Distal cholangiocarcinoma	1 (3)

Continuous variables with a normal distribution are presented as mean ± standard deviation, whereas those without a normal distribution are expressed as median and interquartile range (25th–75th percentile). Categorical variables are reported as numbers of patients, with percentages in parentheses. CT, computed tomography; PVE, portal vein embolization

### Liver volume and iodine concentration measurements

Tables 2 and 3 present the liver volume and iodine concentration measurements of the included patients, respectively. Table 4 presents the interobserver agreements for iodine concentration measurements, generally showing good concordance.

**Table 2:** Results of liver volume measurements

Parameter (n = 39)	Value
TLV (cm^3^)	1247 ± 237
sTLV (cm^3^)	1239 ± 222
Left lobe volume before PVE (cm^3^)	404 (340–452)
Proportion of left lobe volume to TLV (%)	33.2 ± 0.07
Proportion of left lobe volume to sTLV (%)	33.8 ± 0.08
Left lobe volume after PVE (cm^3^)	503 (438–585)
Absolute left lobe volume increase (cm^3^)	114 (89–148)
Hypertrophy rate of left lobe volume increase (%)	30.0 ± 13.3
DH (%)	9.7 ± 4.2
KGR (%/week)	2.9 ± 1.6

Continuous variables with a normal distribution are presented as mean ± standard deviation, whereas those without a normal distribution are expressed as median and interquartile range (25th–75th percentile). DH, degree of hypertrophy; KGR, kinetic growth rate; PVE, portal vein embolization; sTLV, standard total liver volume; TLV, total liver volume

Hypertrophy rate of left lobe volume increase (%) =$\frac{{{\text{Left lobe volume after PVE }} - {\text{Left lobe volume before PVE}}\,}}{{{\text{Left lobe volume before PVE}}}}$× 100

**Table 3:** Results of iodine concentration measurements

Parameter	Value
DECT early-phase iodine concentration (n = 30)	
S2 (mg/mL)	6.03 ± 1.45
S3 (mg/mL)	5.45 ± 1.45
S4 (mg/mL)	5.11 (4.61–6.20)
Mean iodine concentration of S2, S3, and S4 (mg/mL)	5.65 ± 1.41
DECT late-phase iodine concentration (n = 39)	
S2 (mg/mL)	5.76 ± 1.06
S3 (mg/mL)	5.29 ± 1.15
S4 (mg/mL)	4.64 (4.19–5.49)
Mean iodine concentration of S2, S3, and S4 (mg/mL)	5.20 (4.45–5.90)
Contrast volume of the left lobe (cm^3^)	329 (275–378)
DECT early-phase iodine content in the left lobe (mg)	1828 ± 269
DECT late-phase iodine content in the left lobe (mg)	1787 ± 333

Continuous variables with a normal distribution are presented as mean ± standard deviation, whereas those without a normal distribution are expressed as median and interquartile range (25th–75th percentile). DECT, dual-energy computed tomography

**Table 4:** Interobserver agreement for iodine concentration measurements

Parameter	Reader 1	Reader 2	ρ	*P*
DECT early-phase iodine concentration (n = 30)				
S2 (mg/mL)	6.08 (5.30–6.82)	5.77 ± 1.45	0.90	< 0.001
S3 (mg/mL)	5.64 ± 1.56	5.26 ± 1.41	0.94	< 0.001
S4 (mg/mL)	5.60 ± 1.78	5.32 (4.38–5.89)	0.81	< 0.001
Mean iodine concentration of S2, S3, and S4 (mg/mL)	5.84 ± 1.49	5.46 ± 1.36	0.95	< 0.001
DECT late-phase iodine concentration (n = 39)				
S2 (mg/mL)	5.98 ± 1.16	5.53 ± 1.03	0.88	< 0.001
S3 (mg/mL)	5.44 ± 1.22	5.13 ± 1.12	0.88	< 0.001
S4 (mg/mL)	4.77 (4.15–5.61)	4.73 (4.18–5.55)	0.79	< 0.001
Mean iodine concentration of S2, S3, and S4 (mg/mL)	5.35 (4.49–5.97)	5.19 ± 1.00	0.90	< 0.001

Continuous variables with a normal distribution are presented as mean ± standard deviation, whereas those without a normal distribution are expressed as median and interquartile range (25th–75th percentile). DECT, dual-energy computed tomography

Subsequently, patients were categorized into two groups based on a left hepatic lobe hypertrophy rate of ≤ 27.9% and > 27.9% (Table 5), and background factors were compared between the groups; however, no significant differences were found.

**Table 5:** Patient characteristics stratified by left lobe hypertrophy rate after portal vein embolization

	Hypertrophy rate of the left lobe volume (FLR)		
Parameter	≤ 27.9% (n = 20)	> 27.9% (n = 19)	*p*
Age (y)	70 ± 10	73 ± 5	0.309
Male, female	16 (80), 4 (20)	14 (74), 5 (26)	0.749
Body mass index (kg/m^2^)	22.4 ± 4.4	21.3 ± 2.6	0.588
Body surface area (m^2^)	1.63 ± 0.17	1.58 ± 0.18	0.396
Underlying liver disease			
Fatty liver	1 (5)	0 (0)	0.513
Preoperative chemotherapy	5 (25)	1 (5)	0.102
Gemcitabine + Cisplatin + Durvalumab	3 (15)	0 (0)	
Gemcitabine + Cisplatin + S-1	2 (10)	0 (0)	
Gemcitabine + Cisplatin	0 (0)	1 (5)	
Laboratory data			
Total bilirubin level (mg/dL)	0.8 (0.7–1.3)	1.0 ± 0.5	0.411
Albumin level (g/dL)	3.6 ± 0.4	3.5 (3.3–3.8)	0.569
Platelet count (× 10^3^/μL)	198 ± 77	206 (170–328)	0.879
Prothrombin time (%)	99.1 (92.5–104.1)	101.1 ± 7.5	0.336
Interval between PVE and volumetric CT (d)	21.5 (19.5–27.5)	27.1 ± 8.8	0.204
TLV (cm^3^)	1310 ± 239	1181 ± 222	0.175
sTLV (cm^3^)	1275 ± 210	1202 ± 233	0.396
Left lobe volume before PVE (cm^3^)	414 (379–457)	385 ± 91	0.120
Proportion of left lobe volume to TLV (%)	33.3 ± 0.06	33.2 ± 0.07	> 0.999
Proportion of left lobe volume to sTLV (%)	34.6 ± 0.09	33.0 ± 0.09	0.749
DECT early-phase iodine concentration (n = 30)	(n = 17)	(n = 13)	
S2 (mg/mL)	6.20 ± 1.67	5.80 ± 1.13	0.805
S3 (mg/mL)	5.34 ± 1.68	5.59 ± 1.14	0.650
S4 (mg/mL)	4.82 (4.12–4.82)	5.51 ± 1.11	0.483
Mean iodine concentration of S2, S3, and S4 (mg/mL)	5.66 ± 1.66	5.63 ± 1.04	0.680
DECT late-phase iodine concentration (n = 39)			
S2 (mg/mL)	5.79 ± 1.21	5.72 ± 0.90	> 0.999
S3 (mg/mL)	5.23 ± 1.15	5.28 (4.59–5.57)	0.708
S4 (mg/mL)	4.93 ± 1.21	4.64 (4.46–5.04)	0.857
Mean iodine concentration of S2, S3, and S4 (mg/mL)	5.32 ± 1.12	5.28 (4.86–5.64)	0.667
Contrast volume of the left lobe (cm^3^)	338 ± 87	3.26 (3.06–3.78)	0.667
DECT early-phase iodine content in the left lobe (mg)	1796 ± 297	1869 ± 233	0.432
DECT late-phase iodine content in the left lobe (mg)	1740 ± 314	1837 ± 354	0.531
Final diagnosis			
Perihilar cholangiocarcinoma	17 (85)	17 (89)	0.732
Gallbladder carcinoma	3 (15)	1 (5)	0.316
Distal cholangiocarcinoma	0 (0)	1 (5)	0.298

Continuous variables with a normal distribution are presented as mean ± standard deviation, whereas those without a normal distribution are expressed as median and interquartile range (25th–75th percentile). Categorical variables are reported as numbers of patients, with percentages in parentheses. CT, computed tomography; FLR, future liver remnant; PVE, portal vein embolization; sTLV, standard total liver volume; TLV, total liver volume; DECT, dual-energy computed tomography

**Fig. 6:**
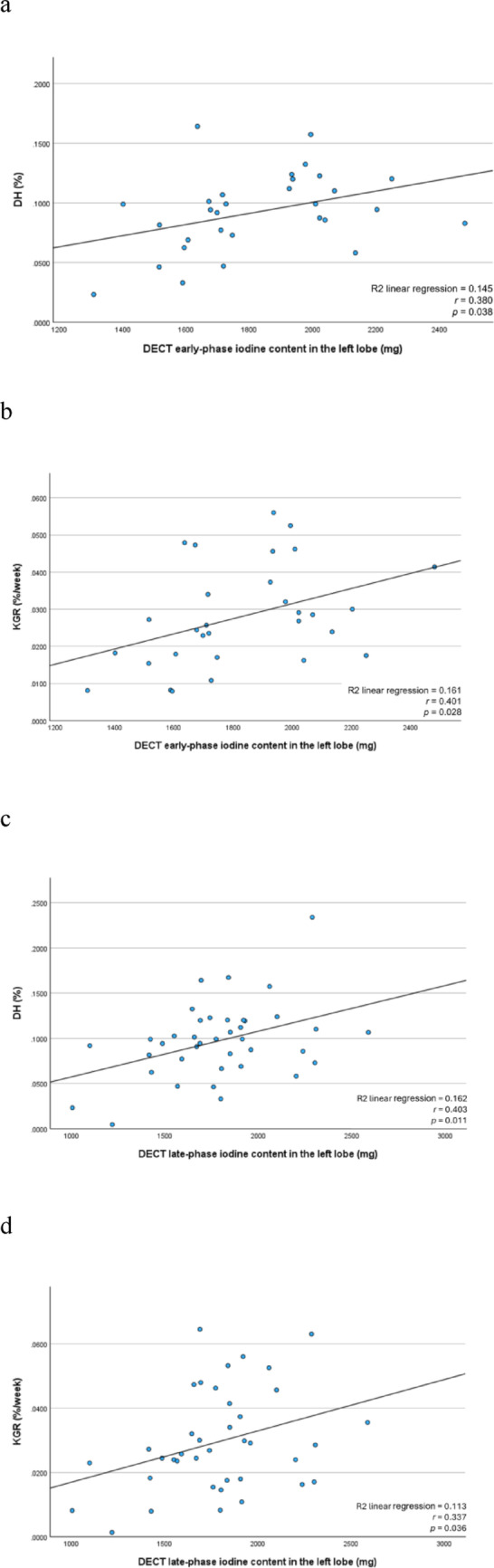
Correlations of the early- and late-phase DECT iodine content in the left hepatic lobe with DH and KGR are summarized. The early-phase iodine content shows significant correlation with **a** DH (*r* = 0.380, *p* = 0.038) and **b** with KGR (*r* = 0.401, *p* = 0.028). The late-phase iodine content demonstrates significant correlations with **c** DH (*r* = 0.403, *p* = 0.011) and **d** KGR (*r* = 0.337, *p* = 0.036). DECT, dual-energy computed tomography; DH, degree of hypertrophy; KGR, kinetic growth rate

The early-phase iodine content of the left lobe on DECT was significantly correlated with both DH (r = 0.380, p = 0.038; Fig. 6a) and KGR (r = 0.401, *p* = 0.028; Fig. 6b), as determined by Pearson’s correlation coefficient.

For the late-phase iodine content of the left lobe, significant correlations were found with both DH (r = 0.403, *p* = 0.011; Fig. 6c) and KGR (r = 0.337, *p* = 0.036; Fig. 6d).

### Post-PVE outcomes

Of the 39 patients enrolled in this study, 31 underwent the planned right hepatectomy, including the caudate lobe after PVE. According to the ISGLS criteria, postoperative liver failure was observed in seven patients, comprising three cases of Grade A and four cases of Grade B; no cases of Grade C were identified. Except for one patient who developed non-occlusive mesenteric ischemia and died on postoperative day 8, all patients were successfully discharged, with a median postoperative hospital stay of 29.5 days. No statistically significant differences were observed in iodine content in the left hepatic lobe according to the presence or absence of postoperative liver failure (Table 6).

**Table 6:** Comparison of iodine content based on the presence or absence of liver failure

	Liver failure		
Parameter	No	Yes	*p*
DECT early-phase iodine content in the left lobe (mg) (n = 23)	1799 ± 216 (n = 18)	1990 ± 474 (n = 5)	0.213
DECT late-phase iodine content in the left lobe (mg) (n = 30)	1851 ± 335 (n = 23)	1617 ± 294 (n = 7)	0.102

DECT, dual-energy computed tomography

The reasons for not proceeding with liver resection in the remaining eight patients were as follows: newly identified distant metastases after PVE in three patients, intraoperative detection of peritoneal dissemination in three patients, deterioration of performance status due to cholangitis after PVE in one patient, and insufficient hypertrophy of the FLR in one patient.

## Discussion

This study investigated the feasibility and clinical utility of a novel technique involving the direct injection of iodinated contrast medium into the portal vein after PVE, followed by a quantitative assessment of iodine distribution in the FLR using DECT, to facilitate risk stratification and increase preoperative planning safety for cases requiring extensive hepatectomy. Total iodine content in the FLR showed moderate positive correlations with both DH and KGR. Specifically, early- and late-phase iodine content was correlated with DH (r = 0.380, *p* = 0.038 and r = 0.403, *p* = 0.011, respectively) and KGR (r = 0.401, *p* = 0.028 and r = 0.337, *p* = 0.036, respectively). These results indicate that higher iodine levels in the FLR are associated with greater subsequent hypertrophy, suggesting that DECT-based iodine quantification may reflect underlying portal perfusion physiology. However, given the modest strength of correlation, the predictive capability of iodine content for FLR hypertrophy remains limited.

Various imaging modalities and biochemical biomarkers have been investigated to predict postoperative liver failure and FLR hypertrophy. Widely used indicators include the ALBI score [21, 22], 99mTc-mebrofenin hepatobiliary scintigraphy [23], Gd-EOB-DTPA–enhanced MRI [24, 25], abdominal ultrasonography [26, 27], and, more recently, portal 4D flow MRI [28]. PVE induces hepatic regeneration by occluding the portal venous inflow to the lobe scheduled for resection and redistributing blood flow toward the contralateral FLR. This hemodynamic alteration is generally considered the primary driver of liver regeneration. However, to our knowledge, no prior study has attempted direct injection of iodinated contrast through a portal catheter followed by DECT-based quantification of portal perfusion. Thus, the present approach can be regarded as novel.

We initially hypothesized that increased portal inflow to the FLR after PVE would result in dilution of the contrast medium and accelerated washout via hepatic venous outflow, thereby leading to reduced iodine concentration. In this model, a decrease in FLR iodine content was expected to serve as a surrogate marker of increased portal perfusion and, ultimately, to correlate with hypertrophy indices such as DH and KGR. Contrary to this assumption, our results demonstrated that total iodine content was positively correlated with both DH and KGR. This finding suggests that early hypertrophy after PVE cannot be explained solely by increased portal inflow. Studies using Doppler ultrasonography and 4D flow MRI have shown that FLR hypertrophy correlates more strongly with hemodynamic changes observed one or several days after PVE rather than immediately after the procedure. This temporal discrepancy supports the notion that FLR hypertrophy depends on mechanisms beyond simple redistribution of blood flow. In addition, Mohkam et al. [29] reported that an increase in hepatic venous pressure gradient after PVE was associated with FLR hypertrophy, suggesting that portal hypertension may contribute to delayed iodine clearance and retention within the hepatic parenchyma. Indeed, portal pressure elevation after PVE has been documented [30, 31], and Qiu et al. [32] demonstrated that impaired iodine clearance assessed by DECT in patients with cirrhosis reflects portal hypertension and correlates with the risk of variceal bleeding. Collectively, these findings indicate that increased iodine retention in the FLR likely reflects complex hemodynamic changes, including both redistribution of portal flow and elevation of portal pressure. The total iodine content observed in this study may therefore reflect not only redistribution of portal flow but also additional parenchymal changes, such as the effects of elevated portal pressure. Nevertheless, because portal pressure was not directly measured in this study, its contribution could not be quantified.

From a technical perspective, our approach also carries significant advantages. A conventional CT approach for assessing hepatic parenchymal enhancement requires paired pre- and post-contrast scans with subtraction of attenuation values. In contrast, DECT generates quantitative iodine maps from a single post-contrast acquisition, avoiding misregistration from varying breath-holds and reducing patient burden owing to the requirement of only a single breath-hold. Nakayama et al. [13] reported superior reproducibility and diagnostic performance for iodine density quantification with DECT than those achieved with conventional HU-based measurements for the assessment of liver fibrosis. In this study, we used a 320-row detector CT in wide-volume (non-helical) mode, which minimizes motion artifacts relative to helical scanning and improves image uniformity [32, 33]. Moreover, unlike peripheral intravenous administration, direct portal venous injection delivers contrast directly to the target organ with minimal first-pass systemic mixing, shortening transit time and making the enhancement less susceptible to interindividual differences in systemic hemodynamics (e.g., cardiac output), circulation time, or body habitus. Notably, FLR iodine content significantly correlated with hypertrophy indices in both early and late phases, suggesting that this method provides predictive information with limited dependence on scan timing. In addition, it can be performed in the same session as PVE without requiring additional examinations, offering practical clinical benefits. However, the observed correlations were only moderate, and further refinement—including optimization of imaging timing and analytic methods—will be necessary before iodine content can be established as a reliable predictor of FLR hypertrophy.

This study has some limitations. First, this was a single-center study with a small sample size. Second, DECT imaging was not performed before PVE due to concerns about radiation exposure and the absence of any previous reports on this technique, which involves inserting a catheter into the portal vein and administering contrast medium during DECT imaging of the liver. Nevertheless, given the promising results, future studies incorporating pre-PVE DECT imaging would be feasible. Third, setting up a control group was challenging due to the invasive nature of the protocol (i.e., catheter insertion into the portal vein). Fourth, the study cohort consisted solely of patients with biliary duct carcinoma. However, most patients did not have chronic liver disease, allowing a more direct evaluation of the PVE effects, and the absence of large liver masses in biliary carcinoma cases facilitated the measurement of liver volume. Fifth, the study also focused exclusively on cases of right PVE, which was appropriate for investigating FLR hypertrophy. Sixth, some variability existed in the timing of the post-PVE CT imaging for liver volume measurement due to individual preoperative conditions and scheduling constraints; however, the interquartile range of 21–30 days suggested that this variability was not excessive. Finally, the proposed approach can only be implemented using a hybrid interventional radiology/CT system equipped with DECT, which limits its availability to specialized centers.

In conclusion, this study demonstrated that iodine content measured by DECT after PVE was moderately correlated with subsequent FLR hypertrophy. However, given the limited strength of this correlation, the present findings do not establish predictive ability. Rather, DECT-based iodine quantification may provide complementary information on portal perfusion and hepatic regenerative capacity. Further investigations with larger cohorts and refined imaging protocols are warranted to clarify its potential role as an imaging biomarker in clinical practice.
